# Supplementary material for: A semiconducting layered metal-organic framework magnet
Source: Nat Commun. 2019 Jul 22;10:3260. doi: 10.1038/s41467-019-11267-w (PMC6646349; doi:10.1038/s41467-019-11267-w)
Supplement: Supplementary file 1 — Supplementary Information [file 41467_2019_11267_MOESM1_ESM.pdf]

A Semiconducting Layered Metal-Organic Framework Magnet

Yang et al

Supplementary Methods

Materials

All the starting materials were purchased from commericial suppliers, such as Sigma-Aldrich, TCI and abcr GmbH. Unless stated, all the chemicals were used directly without further purification. The reactions were performed with the standard vacuum-line and Schleck techniques under nitrogen. Colum chromatography was performed using the silica gel.

Monomer synthesis

(2,3,9,10,16,17,23,24-octahydroxy phthalocyaninato) Metal [II] (***PcM-OH$_8$***, ***M= Zn, Fe; 3***): The synthesis of **PcM-OH$_8$** (**M= Zn, Fe**; ***3***) was carried out according to the previous reported literatures with minor modification.[1,2]

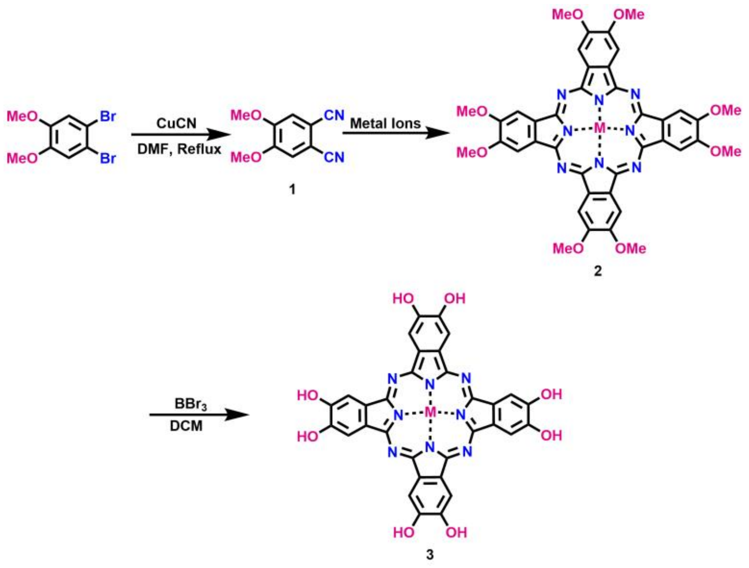

1,2-dicyano-4,5-dimethoxybenzen (***1***): In a 250 mL flask, *1,2-dibromo-4,5-dimethoxybenzen* (10 g, *1 eq*) was added together with copper cyanide (CuCN, 18.18 g, *6 eq*) in 140 mL DMF and then heated at 160 ºC for 4 h. After cooling down to r.t., the reaction mixture was stirred in 340 mL ammonium hydroxide solution (~25% v/v) under air atmosphere overnight. Then, the blue precipitate was collected by filtration and washing with diluted ammonium solution and water, separately. Finally, the obtained olive-green solid was sonicated with 500 mL acetone. The white crude powder extracted from the acetone solution was further

purified through the silica gel column with dichloromethane and iso-hexane. Yield: ~ 42%. ^{1}H NMR ($CHCl_3$): δ 7.10 (s, 2 H, Ar H); δ 3.04 (s, 6 H, OCH_3).

(2,3,9,10,16,17,23,24-octamethoxy phthalocyaninato) Zinc [Zn] (***PcZn-OMe$_8$, 2***): ***1,2-dicyano-4,5-dimethoxybenzen*** (***1***, 0.65 g, 3.46 mmol) and zinc(II) acetate (0.2 g, 1.09 mmol) together with 0.49 g urea were stirred in 32.5 mL 1-pentanol at 150 ºC for 2 days under the argon atmosphere. After cooling to room temperature, the mixture was added with 25 mL H_2O, filtered, and washed with acetone. The obtained green sample was vacuum dried for the next step. Yield: ~72%. ^{1}H NMR (TFA): δ 7.73 (*s*, 8H), 4.29 (*s*, 24H). MS (MALDI-TOF): calculated for $C_{40}H_{32}N_8O_8Zn$: 816.1635 (100%), found: 816.3437 (100%).

(2,3,9,10,16,17,23,24-octamethoxy phthalocyaninato) Metal [Fe] (***PcFe-OMe$_8$, 2***): ***1,2-dicyano-4,5-dimethoxybenzen*** (***1***, 1.0 g, 5.30 mmol) and iron (II) acetate (0.3 g, 1.29 mmol) together with 0.75 g urea were put in a 100 mL two-neck flask under argon. After 50 mL 1-hexanol was added, the whole system was degassed with freeze-pump-thaw methods and then heated at 160 ºC for 2 days under argon atmosphere. After cooling to r.t., the whole mixture was treated with degassed water (40 mL). The obtained precipitate was filtered and washed with acetone for 3 times, after which the dark purple product was vacuum dried for the next step. Yield: ~60%. MS (MALDI-TOF) calculated for $C_{40}H_{32}N_8O_8Fe$: 808.1693 (100%), found: 808.1988 (100%).

(2,3,9,10,16,17,23,24-octahydroxy phthalocyaninato) Metal [Zn] (***PcZn-OH$_8$, 3***): PcZn-OH_8 (0.51 g, 0.62 mmol) and boron tribromide (BBr_3, 2.5 mL, 25.8 mmol) were added to 30 mL dried CH_2Cl_2 at room temperature. The whole mixture was stirred for 4 days under argon atmosphere, after which 23 mL MeOH was added slowly. The solid residue was centrifuged and washed with MeOH for 3 times and the green product PcZn-OH_8 was dried under vacuum. Yield: 73%. ^{1}H NMR (pyridine-d_5): δ 9.57 (s, 8H), 6.79 (s, 8H). MS (MALDI-TOF): calculated for $C_{32}H_{16}N_8O_8Zn$: 704.0383 (100%), found: 704.0089 (100%).

(2,3,9,10,16,17,23,24-octahydroxy phthalocyaninato) Metal [Fe] (***PcFe-OH$_8$, 3***):

The procedure for the synthesis of PcFe-OH_8 was the similar with that of PcZn-OH_8. Yield: 65%. MS (MALDI-TOF): calculated for $C_{32}H_{16}N_8O_8Fe$: 696.0441 (100%), found: 696.0640 (100%).

General Characterization

^1H NMR and ^{13}C NMR spectra were recorded on Bruker AC 300 P using the deuterated solvent ($CDCl_3$, pyridine-d_5) with the tetramethylsilane as the internal reference. MALDI-TOF spectrometry analysis were performed on a Bruker Autoflex Speed MALDI TOF MS (Bruker Daltonics, Bremen, Germany) with trans-2-[3-(4-tert-Butylphenyl)-2-methyl-2-propenylidene] malononitrile as the matrix. Ultraviolet-Visible (UV-vis) spectra were recorded on an Agilent Cary 5000 UV-VIS-NIR spectrophotometer by using 10 mm optical-path quartz cell at room temperature. The cyclic voltammetry (CV) experiment was performed with tetra-n-butyl-ammoniumhexafluorophosphate ($TBAPF_6$, 0.1M) as supporting electrolyte at 298 K at the scan rate 100 mV/s. The conventional three electrode cell was used with a glassy carbon working electrode (surface area of 0.3 mm^2) and a platinum wire as the counter electrode. Fourier transform infrared spectroscopy (FTIR) was performed on a Spectrum 100 (Perkin Elmer, Inc., USA) spectrometer with a scan range of 4000-400 cm^{-1}. X-ray diffraction (XRD) patterns were recorded on an X-ray diffractometer (D/max-2200/PC, Rigaku) using Cu-Kα radiation (λ = 0.15418 nm) at 40 kV. The data of X-ray absorption near edge structure (XANES) spectra and extended X-ray adsorption fine structure (EXAFS) spectra were collected at room temperature in transmission mode at beamline BL14W1 and BL15U1 of the Shanghai Synchrotron Radiation Facility (SSRF, China). The scanning electron microscopy (SEM) and the transmission electron microscopy (TEM) images were recorded on a field emission scanning electron microscope (FESEM, Zeiss Gemini 500) and a high-resolution transmission electron microscope (HRTEM, JEM-2100, JEOL, Japan), respectively. Thermal gravimetric analysis (TGA) was performed using a TA-Instruments Q5000IR thermos gravimetric analyzer in flowing (100 mL/min) nitrogen atmosphere. Nitrogen sorption isotherms were measured on a BELSORP

adsorption analyzer at liquid nitrogen temperature, and the surface area was calculated based on the adsorption curve according to the Brunauer-Emmett-Teller (BET) theory. Nitrogen sorption measurements were conducted at 77 K on a Quantachrome volumetric analyser. Before the measurement, the powder sample was treated in a supercritical CO_2 dryer for 5 days, and then activated at 80 °C overnight. The X-ray photoelectron spectroscopy (XPS) spectra were acquired using a Kratos Axis UltraDLD spectrometer (Kratos Analytical-A Shimadzu Group Company) with a monochromatic Al-Kα source (1486.6 eV). Magnetic properties were studied by employing a Quantum Design MPMS XL Superconducting Quantum Interference Device (SQUID) magnetometer equipped with a different field option (100 Oe, 1T).

THz measurements and calculation

THz Time Domain Spectroscopy (TH_Z-TDS) measurements were realized by employing a titanium sapphire laser (800 nm, 40 fs FWHM, 1 kHz repetition rate). The 800 nm laser output was frequency doubled to 400 nm in a BBO crystal (acting as pump source) and the single-cycle THz probe pulses (bandwidth of ~2 THz) were generated by optical rectification (0.5 mm thick ZnTe crystal). The transmitted THz field was detected with in a second ZnTe by the electro-optic effect. By mechanical delays, both the carrier dynamics and the frequency-resolved conductivity could be obtained. The sample was drop-casted onto fused silica substrates and measured under nitrogen environment. The phase-sensitive detection of the THz pulses allows estimating the complex valued conductivity σ(ω) of the photo-excited sample. From the resolved photoconductivity and employed pump fluence, the mobility μ(ω) of the carriers can be estimated by normalizing the conductivity by the employed pump fluence F_p: $\mu(\omega) = \frac{\sigma(\omega)}{eF_p}$,, where e denotes the elementary charge. This approach provides a good (lower) estimate of the mobility by assuming that each absorbed photon photo-generate one electron-hole pair.

The line shape of the complex valued conductivity for the K_3Fe_2[PcFe-O_8] can be well fitted by the Drude-Smith model. This model has been widely employed to

phenomenologically describe the transport of carriers in nanostructured systems. It assumes that carriers move freely with an average scattering time τ. Upon a scattering event a carrier changes its velocity so that the fraction c of the original velocity is retained in the original direction ($c < 0$ means that charges experience backscattering and $c=0$ leads to isotropic scattering and the traditional Drude free-carrier model). The carrier mobility then reads according to equation in Drude-Smith model (Methods in main text).

Modeling and electronic structure

Density functional theory (DFT) calculations were carried out using the Vienna ab-initio Simulation Package (VASP)[3,4] version 5.4.1. The electronic wave-functions were expanded in a plane-wave basis set with a kinetic energy cutoff of 500 eV. Electron-ion interactions were described using the projector augmented wave (PAW) method.[5,6] Generalized gradient approximation (GGA)[7] of the exchange-correlation energy in the form of Perdew-Burke-Ernzerhoff (PBE)[8] was applied. We used DFT+U approach[9] to describe the localized *d*-orbitals of Fe ions. The effective Coulomb (U) and exchange (J) terms were set to 4 and 1 eV, respectively. Such combination of U and J were already successfully applied for very similar systems.[10] The K_3Fe_2[PcFe-O_8] MOF monolayer was modeled by adding a large vacuum space of 10 Å in the direction normal to the monolayer. Monkhorst-Pack[11] Gamma-centered grid with 6×6×1 dimension was used for k-point sampling of the Brillouin zone. In the computational protocol for the 3D stacking of the studied MOF the k-point grid dimension was changed to 2×2×6 and Grimme-D2 correction[12] was applied.

The unit cell of the K_3Fe_2[PcFe-O_8] monolayer is composed of 3 Fe ions, one in the phthalocyanine (PC) unit and two Fe ions in the iron-bis(dihydroxy) linkages. Three K^+ counter ions were added in the unit cell to ensure that we have one Fe^{3+} and two Fe^{2+} ions in the nit cell of the monolayer.

The structure of the monolayer was first fully optimized in spin nonpolarized calculation and then three different magnetizations of the Fe ions in the monolayer were tested using MAGMON tag in VASP. In the first case, all Fe ions in the unit cell

have parallel spins (high spin). In the second case only one of the Fe ions in the iron-bis(dihydroxy) linkages and the Fe ion in the phthalocyanine (Pc) ring have parallel spins, while in the third case the spin of the Fe ion in the Pc unit is opposite to the spin of the two Fe ions in the iron-bis(dihydroxy) linkages (low spin). The later one is energetically most favorable. From the band structure of the monolayer models one can see that the monolayer is a semiconductor wit narrow band gap of 0.12 eV.

For the 3D structure of $K_3Fe_2[PcFe\text{-}O_8]$, three types of layer-stacking were considered (AA, AA-serrated, and AB). The unit cell for the 3d structure contains two layers in *c* direction for AA-serrated, and AB stacking and one layer for AA stacking. For all of them a full cell relaxation was performed, using ISIF = 3 tag in VASP. The relative stability and the corresponding PXRD patterns for each stacking mode were compared with the experimental one and it was found that the PXRD patterns of AA and AA-serrated stacked layers fit best to the experiment. We focused our discussion on AA-serrated stacking because it is energetically more favorable over the AA stacking by 0.28 eV per $K_3Fe_2[PcFe\text{-}O_8]$ unit cell (Fig S9). The magnetic state of the layered 3D structure in AA-serrated stacking was calculated with ferromagnetic and antiferromagnetic arrangement of the spins on the Fe ions in the phthalocyanine ring in a direction normal to the layers. The energy difference between ferromagnetic (FM) and antiferromagnetic (AFM) state, $E_{ex} = E_{AFM} - E_{FM}$, is 300 meV in a favor of the FM state (Fig. S17 shows the spin density of AA-serrated stacking model). From the band structure of the 3D model in AA-serrated stacking mode we found a very narrowband gap of 0.10 eV (Fig. 2c in main text).

Supplementary Figures

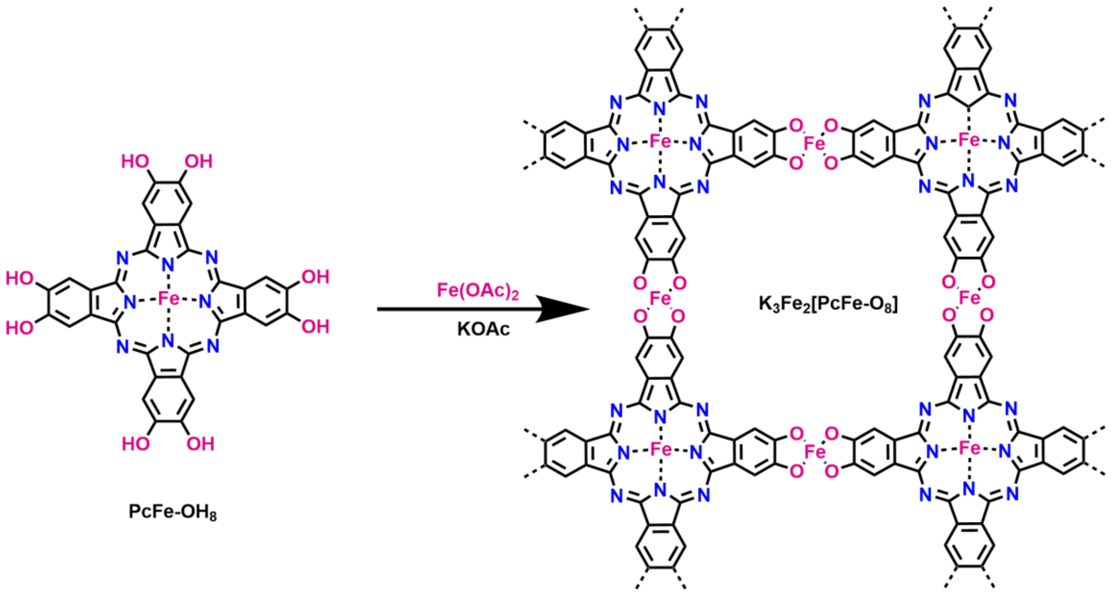

Supplementary Figure 1 | The synthesis and chemical structure of $K_3Fe_2[PcFe\text{-}O_8]$.

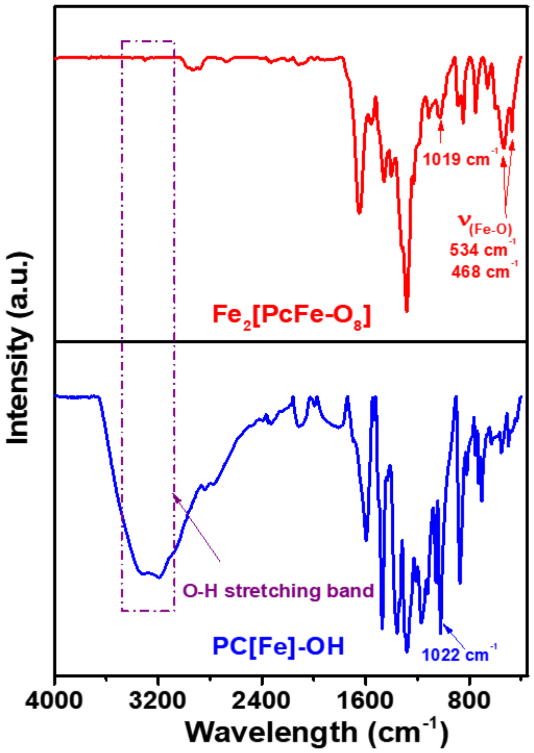

Supplementary Figure 2 | FTIR of monomer PcFe-OH_8 and K_3Fe_2[PcFe-O_8].

Compared to the FTIR spectrum of the monomer, the strongly reduced band O-H stretching band at ~3300 cm^{-1} indicated the successful coordination reaction between the -OH groups and iron ions.[13] Moreover, the newly appeared bands at 534 cm^{-1}, 468 cm^{-1} could be assigned to the O-Fe-O stretching bonds.[14] The peak centered at 1019 cm^{-1} for K_3Fe_2[PcFe-O_8] exhibits an obvious shift to lower energy compared with the C-O stretching at 1022 cm^{-1} for the monomers, which can be induced by the C-O···Fe stretches originating from the electronic perturbation of the σ bond donated by the C-O groups to the Fe atoms.

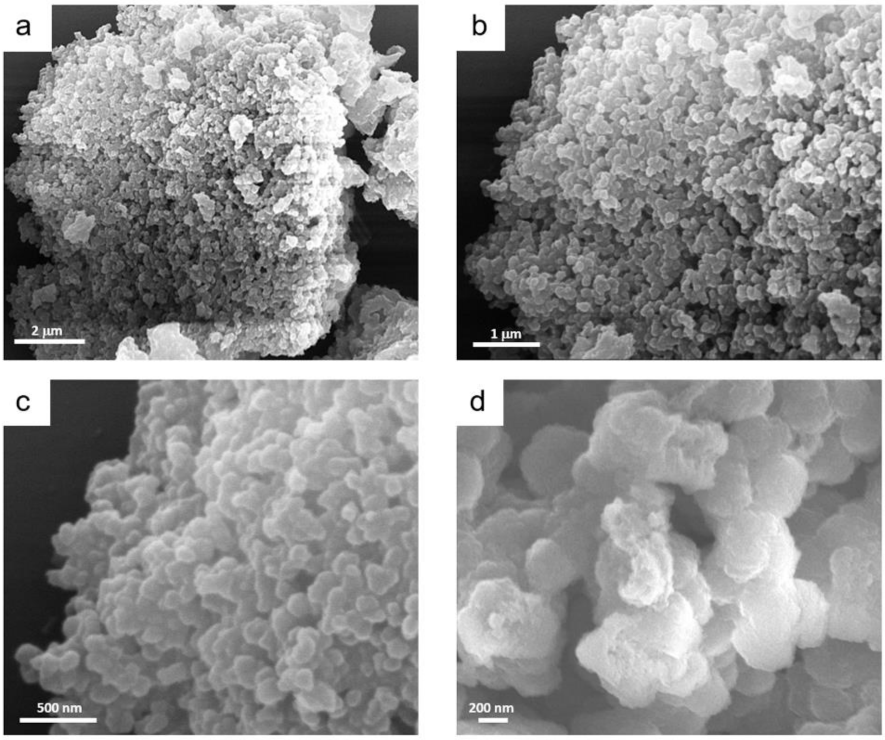

Supplementary Figure 3 | a-d) SEM images of $K_3Fe_2[PcFe\text{-}O_8]$ with different magnification.

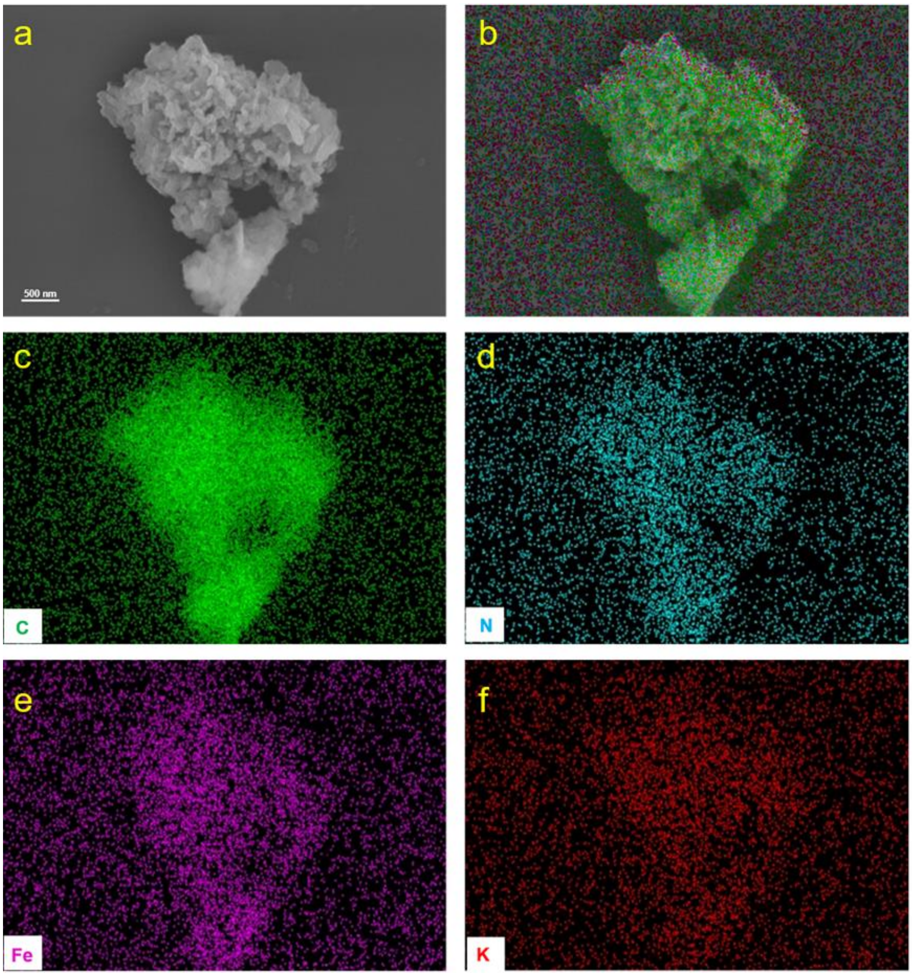

Supplementary Figure 4 | SEM image of $K_3Fe_2[PcFe\text{-}O_8]$ a) and the corresponding elemental mapping images: full mapping image b); c) C; d) N; e) Fe; f) K. With energy-dispersive X-ray spectroscopy, the uniformly distributed C, N, Fe and K elements confirmed the existence of these components in $K_3Fe_2[PcFe\text{-}O_8]$.

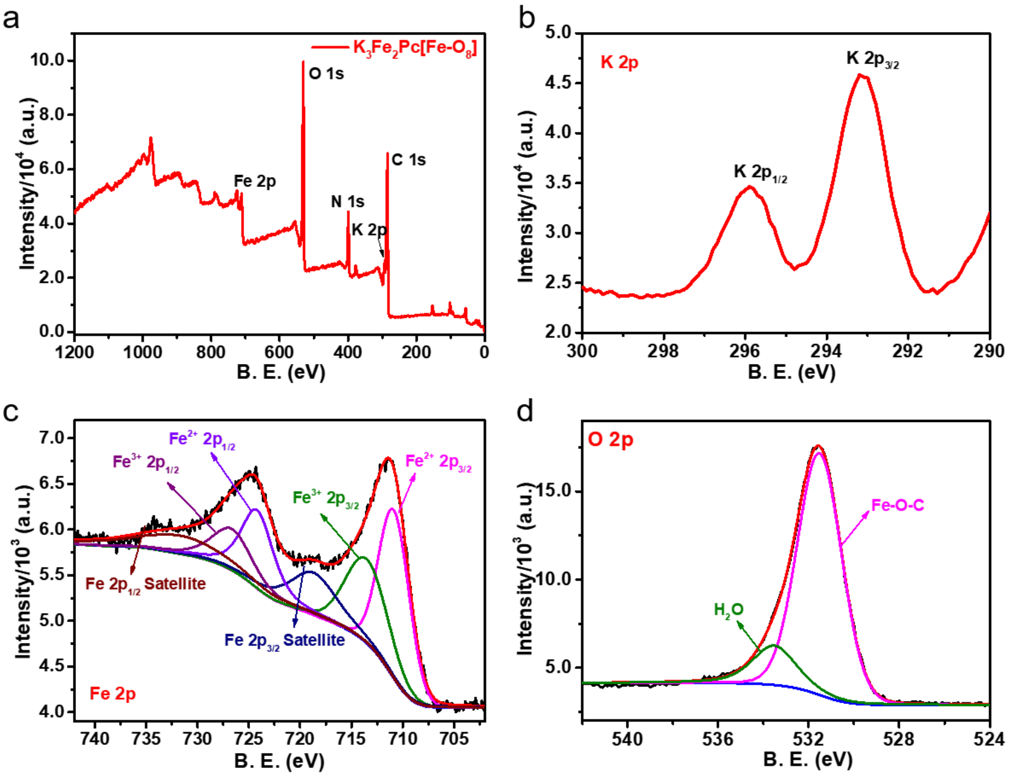

Supplementary Figure 5 | a) XPS survey spectrum of $K_3Fe_2[PcFe\text{-}O_8]$; Corresponding core level spectra of K(*2p*) b); Fe (*2p*) c) and O (*2p*) d). The XPS spectrum of $K_3Fe_2[PcFe\text{-}O_8]$ confirmed the existence of C, N, O, Fe and K (Fig. S5a and 5b). Fe *2p* core level spectrum in Fig. S5c consists of the $Fe_{(II)}$ $2p_{3/2}$ peak at 710.8 eV, $Fe_{(III)}$ $2p_{3/2}$ peak at 713.4 eV, $Fe_{(II)}$ $2p_{1/2}$ peak at 724.2 eV, $Fe_{(III)}$ $2p_{1/2}$ peak at 726.6 eV. The satellite peaks of Fe $2p_{3/2}$ and $2p_{1/2}$ lie at 719.7 eV and 732.5 eV, respectively.[15] The O *1s* core level spectrum (Supplementary Fig. 5d) found the Fe-O-C peak at 531.5 eV, and the adsorbed H_2O/O_2 peak at 533.5 eV.[16]

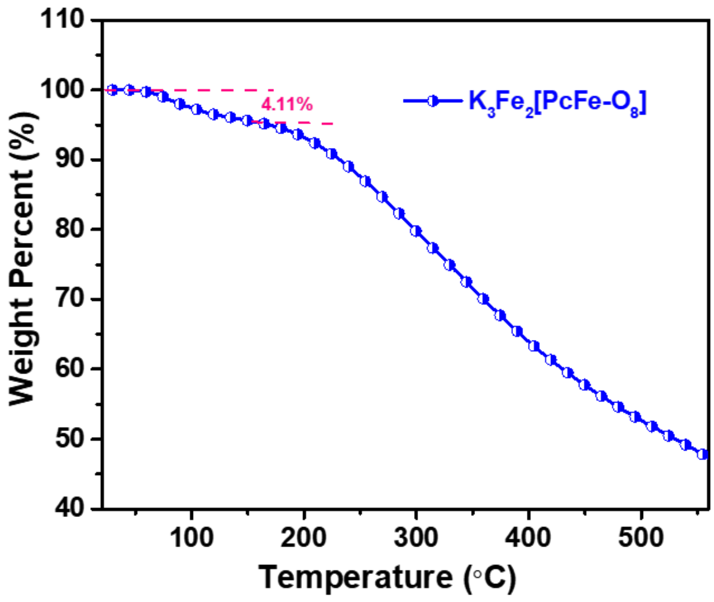

Supplementary Figure 6 | TGA analysis of K_3Fe_2[PcFe-O_8] under nitrogen atmosphere.

As shown in Supplementary Fig. 6, the TGA spectrum shows that the K_3Fe_2[PcFe-O_8] starts to desolvate H_2O at 353 K (80 ºC). A weight loss of 4.11% is an evidence of the coordinated water in K_3Fe_2[PcFe-O_8]. This result implies the existence of ~2 coordination H_2O, leading to a chemical formula for this synthetic layered MOF as K_3Fe_2[PcFe-O_8]•2H_2O. The high-resolution O 2p XPS spectrum (Supplementary Figure 5d) also shows the H_2O peak at 533.5 eV, which is in consistence with the TGA results. In order to check the validity of our K_3Fe_2[PcFe-O_8]·2H_2O formula, we have performed elemental analysis by a combination of inductively coupled plasma optical emission spectrometry (ICP-OES) and C, H, O and N combustion method; the results (in percentage) are presented in the table below:

	Fe(%)	C(%)	H(%)	N(%)	K(%)	O(%)
**Found**	17.39	40.16	1.38	11.83	12.13	17.05
**Calculated**	17.57	40.32	1.27	11.75	12.30	16.78

From these estimates, and taking into account of the formula $C_{32}H_{12}Fe_3K_3N_8O_{10}$, we can obtain the following elemental ratios: C/Fe ≈ 32/3; C/H ~ 32/13.2; C/N ≈ 32/8; C/K ≈ 32/3; C/O ≈ 32/10.2, Therefore, the element analysis finally validates a chemical formula as K_3Fe_2[PcFe-O_8]·2.2H_2O ($C_{32}H_{12.4}Fe_3K_3N_8O_{10.2}$) for the layered MOF that we have developed (named as K_3Fe_2[PcFe-O_8] for short).

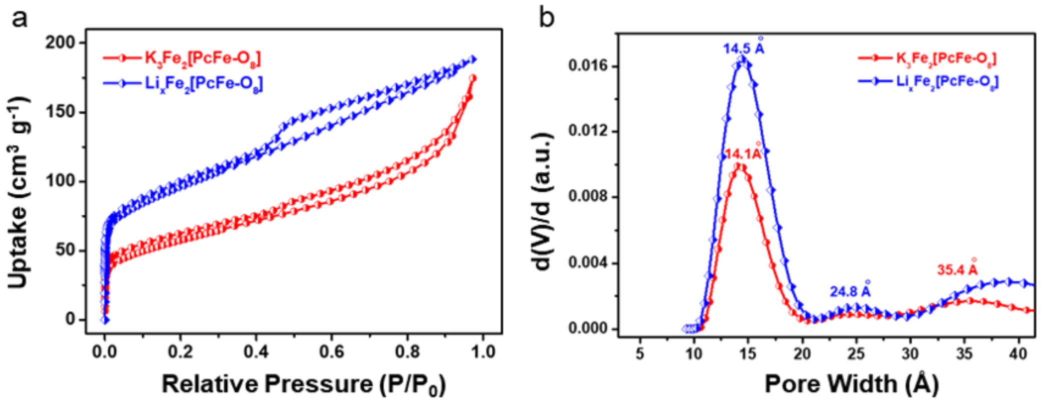

Supplementary Figure 7 | Porous analysis of $K_3Fe_2[PcFe\text{-}O_8]$ (red curves) and $Li_xFe_2[PcFe\text{-}O_8]$ (blue curves). a, N_2 adsorption isotherm measured at 77 K after supercritical CO_2 drying. **b,** Pore size distribution profile calculated from the adsorption data using the DFT model.

The synthesis of $Li_xFe_2[PcFe\text{-}O_8]$ was performed according to the same method for $K_3Fe_2[PcFe\text{-}O_8]$. The average microporous size (~ 1.4 nm) is evident for $K_3Fe_2[PcFe\text{-}O_8]$, which is smaller than the calculated value (~ 1.8 nm), due to the balance K^+ ions in the pores. As contrast, after we changed the K^+ ions by smaller Li^+ ions ($Li_xFe_2[PcFe\text{-}O_8]$), the BET surface area for $Li_xFe_2[PcFe\text{-}O_8]$ was enhanced to be 343 m^2 g^{-1} (Figure a, blue line) and the average microporous pore size was determined as ~1.45 nm (Figure b, blue line), which further revealed the blocking influence of the incorporated counter ions on the porosity.

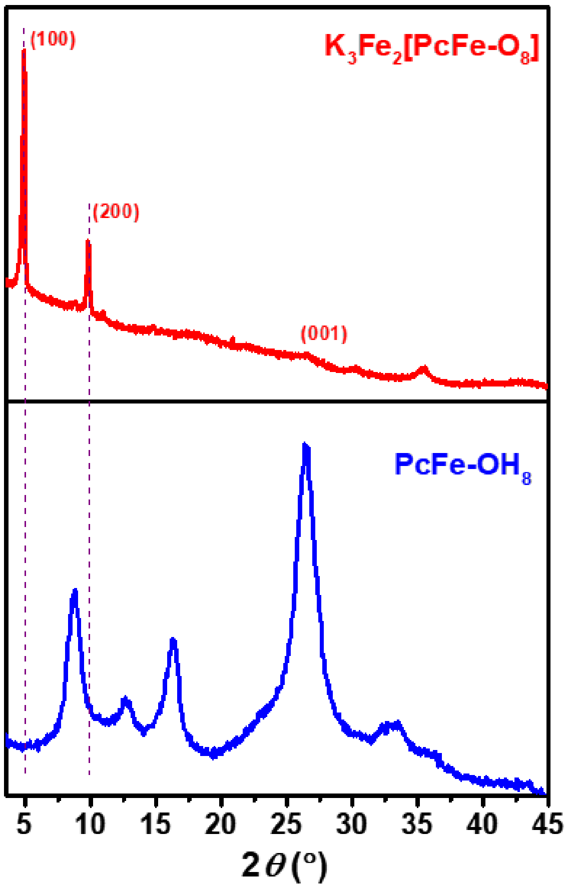

Supplementary Figure 8 | PXRD patterns of monomer PcFe-OH$_8$ and K$_3$Fe$_2$[PcFe-O$_8$] layered MOF.

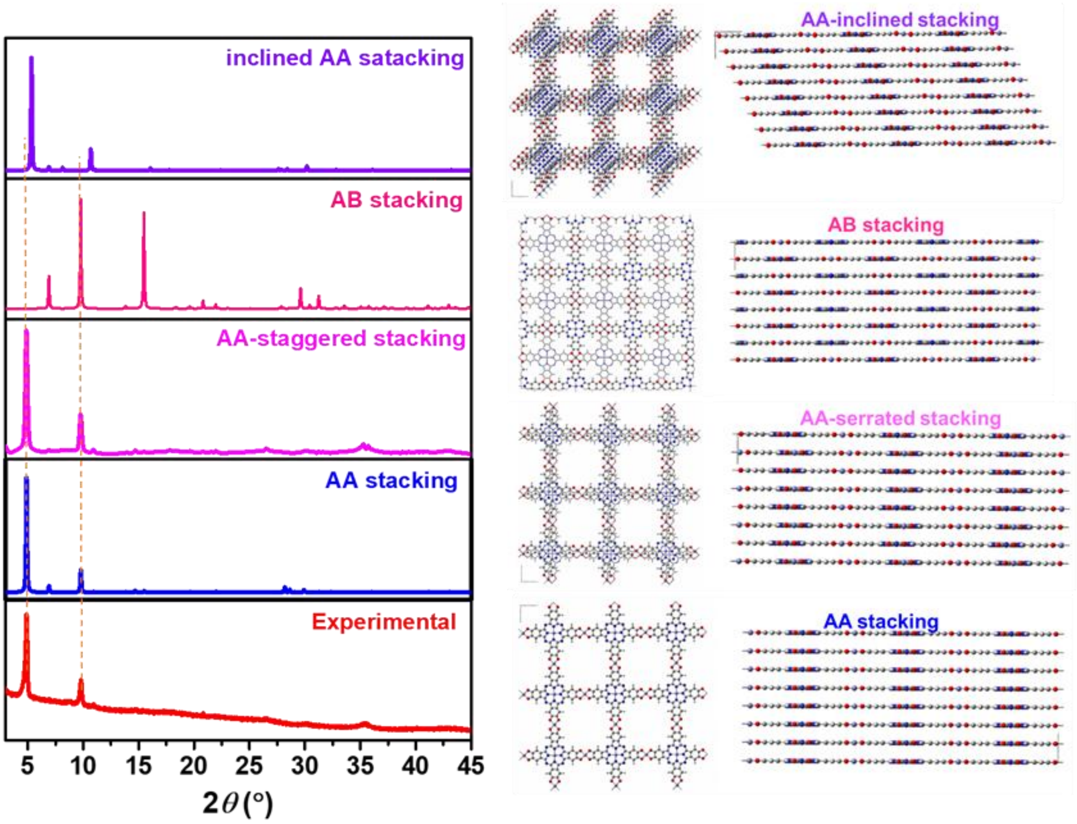

Supplementary Figure 9 | Calculated X-ray diffraction pattern for the layered structures with the AA-inclined, AB, AA-serrated and AA stacking modes compared with the experimental pattern (red) of $K_3Fe_2[PcFe\text{-}O_8]$. These results show that both calculated PXRD patterns from AA stacking and AA-serrated stacking structures agreed well with the experimental result. Besides, the MOF with AA-serrated stacking structure shows more energetic stability by 0.29 eV.

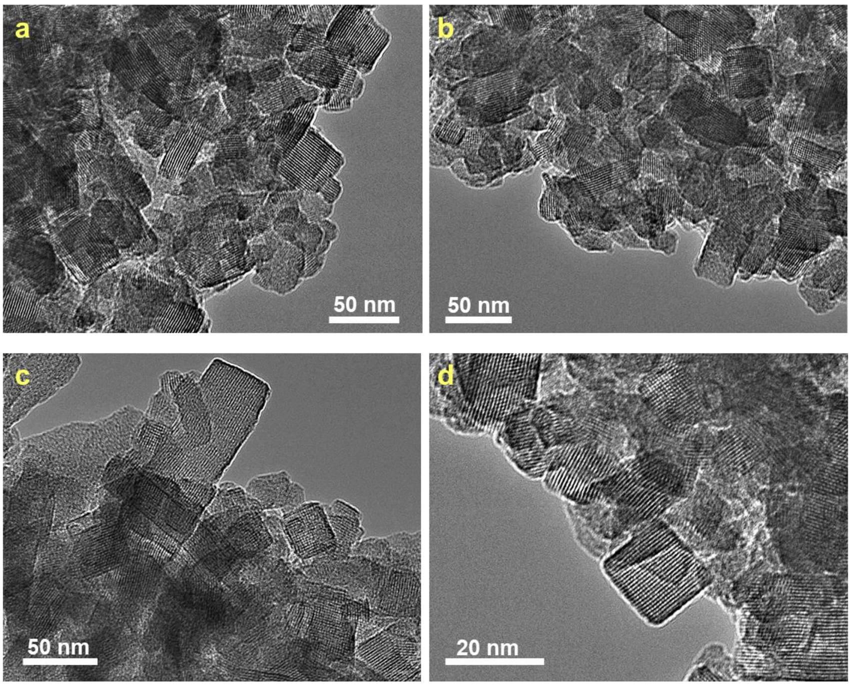

Supplementary Figure 10 | TEM images of $K_3Fe_2[PcFe\text{-}O_8]$ within different regions of the powder samples.

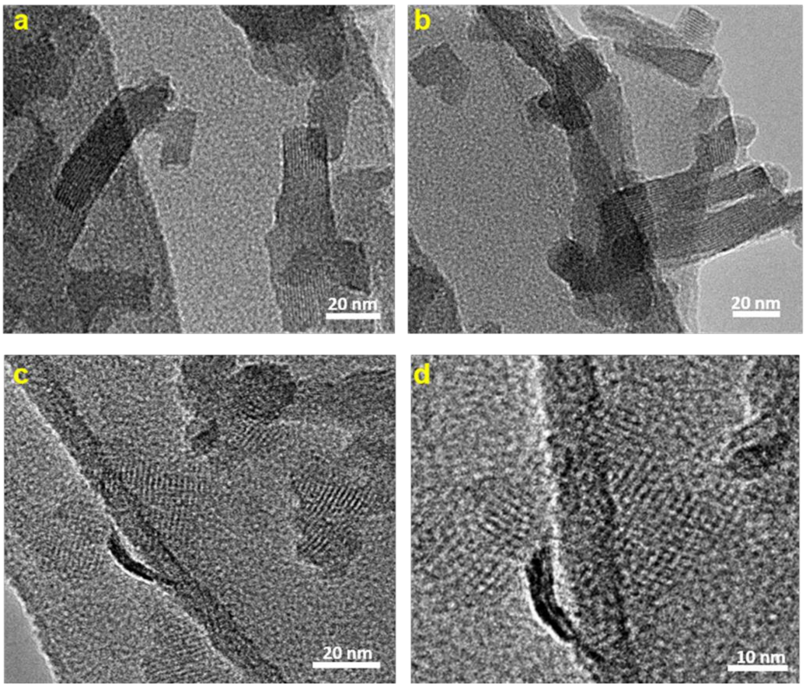

Supplementary Figure 11 | TEM images of K_3Fe_2[PcFe-O_8] with different magnification. TEM results show the rectangle-shaped crystals of K_3Fe_2[PcFe-O_8]. Notes: The crystallinity and the structural determination (such as repeated units, stacking pattern, amorphous structures and impurities) are the key research motivation towards the controlled synthesis of MOFs. As one of the common targets, it remains challenging to synthesize highly crystalline or even single-crystalline layer-stacked MOFs, which can be essential to achieve reliable structure-property relationship based on the pristine sample. Notably, a few successful examples about the magnetic layered MOFs have been recently reported by Coronado et al. (Nat. Chem. 2018, 10, 1001-1007), Clérac et al. (Nat. Chem. 2018, 10, 1056-1061), Harris et al. (J. Am. Chem. Soc. 2015, 137, 15699-15702; J. Am. Chem. Soc. 2017, 139, 4175-4184) and Long et al. (J. Am. Chem. Soc. 2015, 137, 15703-15711; J. Am. Chem. Soc. 2018, 140, 3040-3051). Here in this work, we synthesized a magnetic, polycrystalline, layer-stacked MOF, which comprises of poly-dispersed nanoscale crystallites. It is still difficult to exclude the possible presence of the amorphous phases from the current XRD and local HR-TEM characterizations. Nevertheless, we put our efforts to exclude the possible iron impurities in the resultant K_3Fe_2[PcFe-O_8] by the above spectroscopy studies (Fig. 1, Supplementary Fig. 5). We also acquired a number of TEM images

within different regions of the sample, as shown in Supplementary Fig. 10-11. Based on these TEM images, the rectangle-shaped nanocrystals of $K_3Fe_2[PcFe\text{-}O_8]$ are well visualized. In fact, our work indeed offers a high-quality structural resolution by TEM, which clearly presents nanoscale crystalline domains in $K_3Fe_2[PcFe\text{-}O_8]$ comprising square lattices.

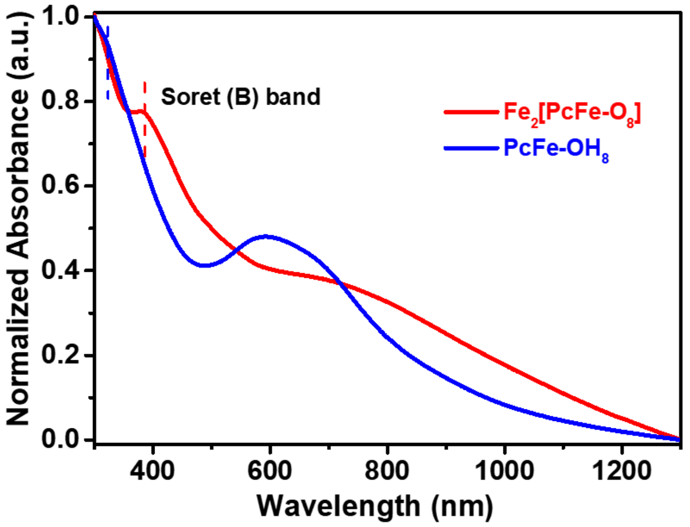

Supplementary Figure 12 | UV/vis spectra of PcFe-OH$_8$ and K$_3$Fe$_2$[PcFe-O$_8$]. Compared with the Soret-band of the monomer at 317 nm, which indicates a typical *π*-*π** transition of the phthalocyanine rings, K$_3$Fe$_2$[PcFe-O$_8$] exhibits obvious red shift of ~ 68 nm, indicating the extended conjugated planes.[17,18]

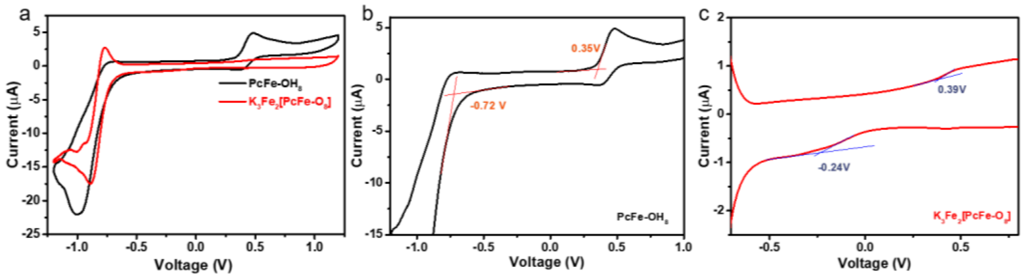

Supplementary Figure 13 | a) CV profiles of PcFe-OH$_8$ and K$_3$Fe$_2$[PcFe-O$_8$] measured in CH$_3$CN at a scan rate of 100 mV·s^{-1}; Magnification of CV profiles for PcFe-OH$_8$ b), K$_3$Fe$_2$[PcFe-O$_8$] c). E_{HOMO}= -E_{ox}- 4.8 eV; E_{LUMO}= -E_{Red}- 4.8 eV; $E_{g, CV}$= E_{HOMO} – E_{LUMO}.

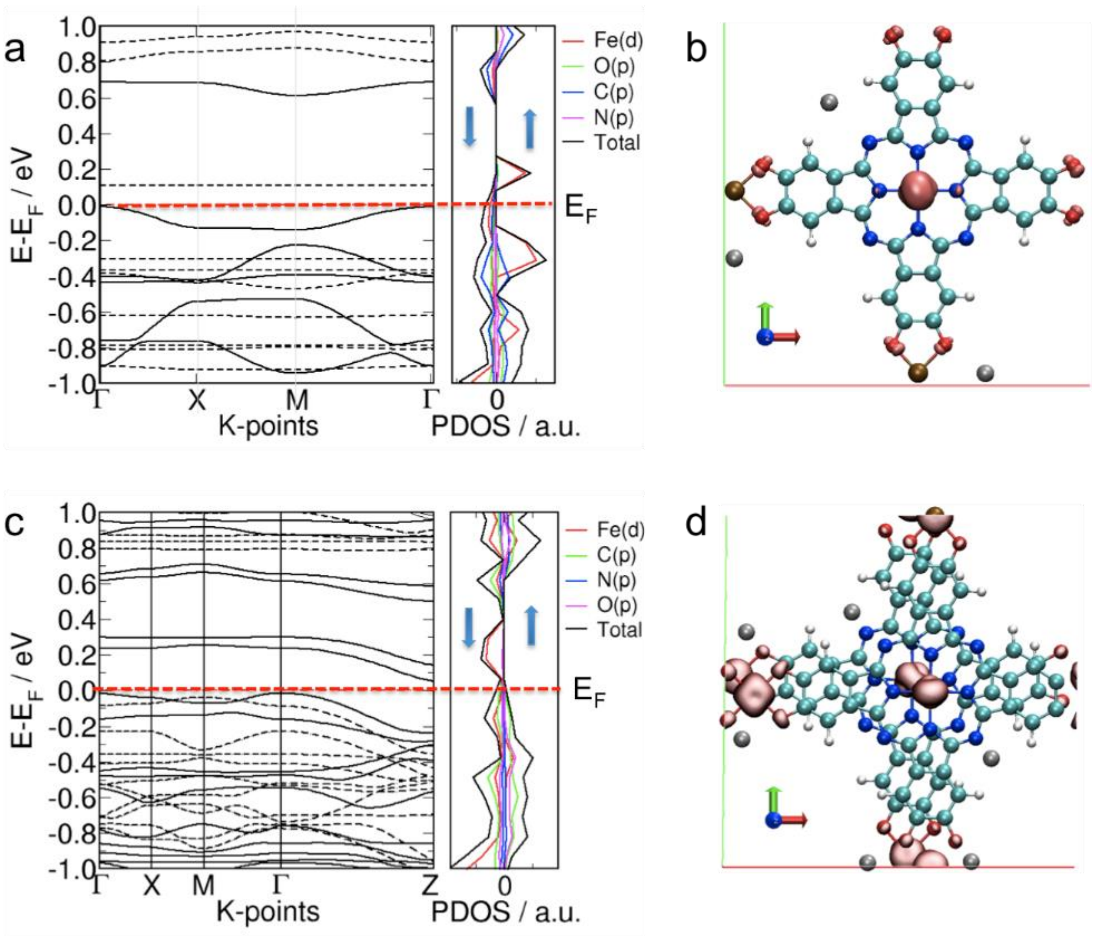

Supplementary Figure 14 | Modelling of the electronic structures of K_3Fe_2[PcFe-O_8]. a, Band structure of a monolayer K_3Fe_2[PcFe-O_8] with GGA+U correction shown on the left panel, dashed lines indicate the bands associated to spin up while the solid lines indicate the bands associated to spin down (the effective Coulomb (*U*) and exchange (*J*) terms reported in the Supplementary Information). The corresponding projected density of states (PDOS) for spin up and spin down are plotted on the right panel for Fe(*d*), C(*p*), O(*p*), and N (*p*) states; **b,** Spin density iso-surface (pink solid iso-surface), at absolute spin-density $|\rho^{\uparrow} - \rho^{\downarrow}| = 0.05$ electrons per Å^3 of a monolayer K_3Fe_2[PcFe-O_8], indicating that the spin density is mainly localized on the Fe ions (light cyan: C; blue: N; red: O; light red: Fe; grey: K^+); **c,** Calculated electronic band structure of multi-layered K_3Fe_2[PcFe-O_8] with AA-serrated stacking mode; **d,** Unit cell of two-layered K_3Fe_2[PcFe-O_8] in AA-serrated stacking mode with ferromagnetic arrangement.

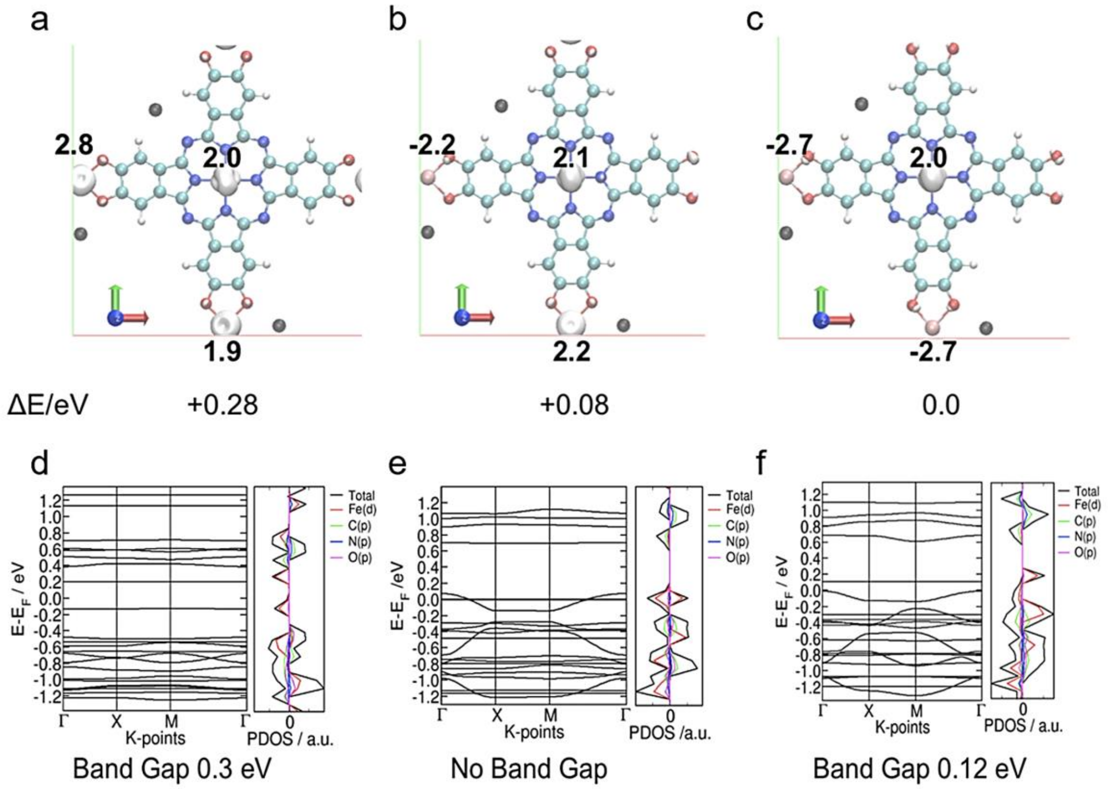

Supplementary Figure 15 | a-c) Different spin density iso-surface and magnetic moment per Fe ion (in μ_B) in monolayer of K_3Fe_2[PcFe-O_8] MOF. The relative stability for each magnetic configuration (*ΔE*) is shown in eV; **d-f) The corresponding band structures are shown below each structure**. Positive value of the magnetic moment per Fe means spin up, while the negative value means spin down. Notably, only the positive value provides the spin density in a K_3Fe_2[PcFe-O_8] unit cell. Besides, the spin of Fe in the phthalocyanine ring is lower than that of Fe in linkage, indicating the different oxidation states of Fe atoms.

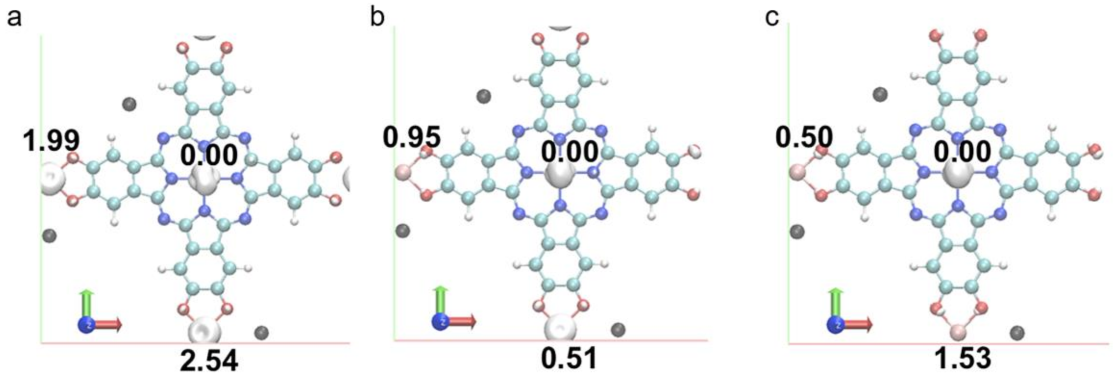

Supplementary Figure 16 | Calculated Fe(*2p*) core level shift per Fe ion. The energy of the Fe(*2p*) core level shift shows the oxidation state of the Fe ion. A reference value was chosen the Fe(*2p*) core level energy of the Fe in the phthalocyanine ring. Positive core level shift means destabilization in the core levels, i.e. reduction of the oxidation state. Thus, it is indicated that the Fe in the ring is always in higher oxidation state than the Fe ions in the linkages, because their Fe(*2p*) levels are higher in energy by 0.5-2.5 eV, which is consistent with XPS result.

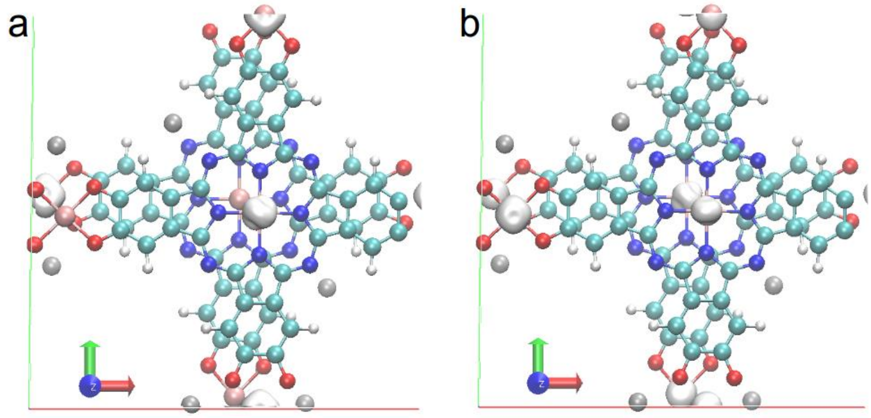

Supplementary Figure 17 | Unit cell of a 3D structure in AA-serrated stacking with antiferromagnetic arrangement a) and ferromagnetic arrangement b) of the spin up electrons. Taking AA-serrated stacking into consideration, the ferromagnetic order between two neighboring layers in a unit cell of bulk $K_3Fe_2[PcFe\text{-}O_8]$ is more energetically favorable by 300 meV than that of antiferromagnetic arrangement.

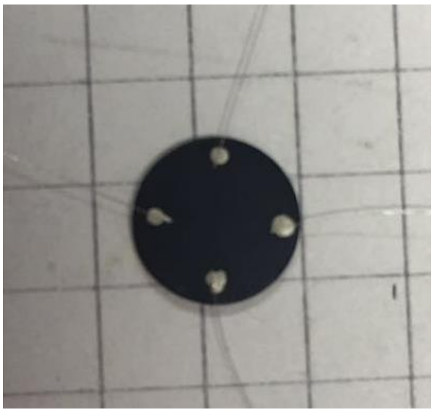

Supplementary Figure 18 | Pressed pellets device of $K_3Fe_2[PcFe-O_8]$ for variable-temperature conductivity measurements by *van der Pauw* method. The powder MOF samples were pressed into pellets at 398 K under vacuum by adding ~1 GPa pressure. The thickness of the pressed pellet was measured as ~0.592 mm.

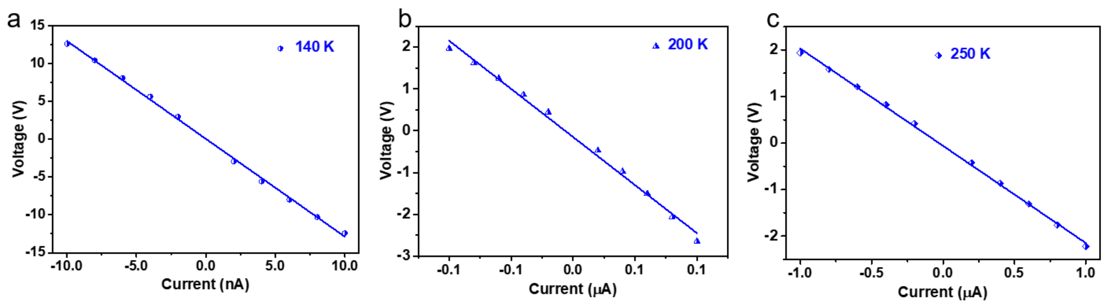

Supplementary Figure 19 | *I-V* curves for $K_3Fe_2[PcFe\text{-}O_8]$ collected at 140 K, 200 K and 250 K display Ohmic responses. Similar responses were observed till the measured limit 350 K.

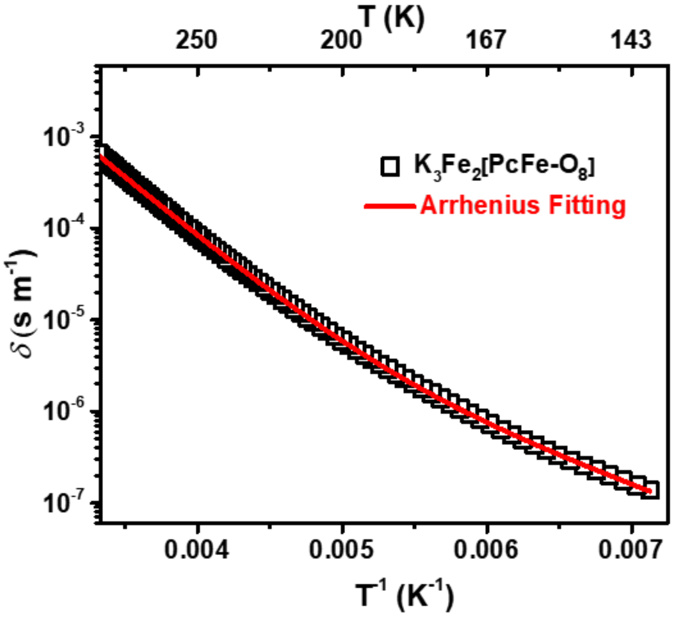

Supplementary Figure 20 | Variable-temperature electrical conductivity of $K_3Fe_2[PcFe-O_8]$ as a function of inverse temperature. The red solid lines show the fits of data to Eq. (1).

To describe a thermally activated conduction process at high and low temperatures, we used a model given in Eq. (1):

$$\sigma = \frac{\sigma_1}{\exp[E_1/k_B T]} + \frac{\sigma_2}{\exp[E_2/k_B T]} \quad (1)$$

where preexponential constants σ_1, σ_2 and activation energies E_1, E_2 are the free parameters. The measured temperature dependent conductivity data fit to this model quite nicely (from 150 K to 300 K). The fitting results indicate two thermally activated contributions to the conductivity of our sample. For $K_3Fe_2[PcFe-O_8]$ MOF, a high temperature activation region with an activation energy ~261 meV (E_1) and a low temperature activation region with an energy ~115 meV (E_2) were observed.

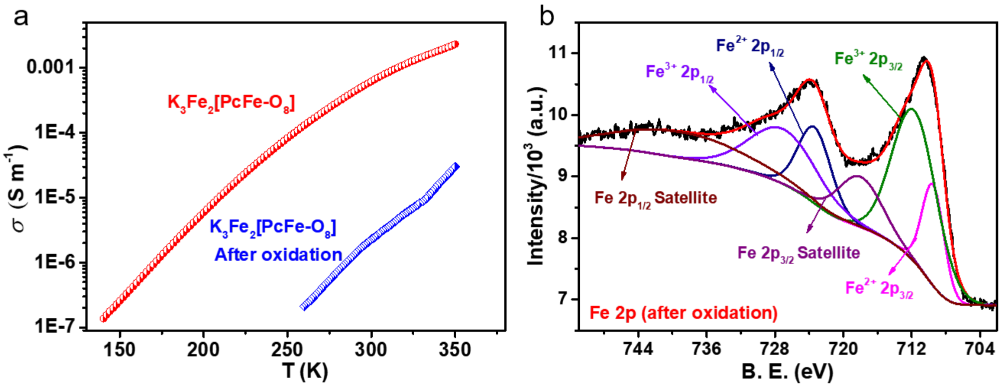

Supplementary Figure 21 | Contrast experiments after air-oxidation. a, Variable-temperature electrical conductivity of $K_3Fe_2[PcFe\text{-}O_8]$ before (in red) and after oxidation (in blue) through *van der Pauw* method; **b,** Core level spectrum of Fe (*2p*) of $K_3Fe_2[PcFe\text{-}O_8]$ after oxidation.

The mixed oxidation states of two Fe ions contribute a lot to the conductivity of MOF materials. Therefore, we remeasured the conductivity of $K_3Fe_2[PcFe\text{-}O_8]$ after oxidation by air for one week. As shown in Supplementary Figure 22a, the conductivity after oxidation is 2-3 orders of magnitude lower than before (250-350 K). At 350 K, the conductivity of $K_3Fe_2[PcFe\text{-}O_8]$ after oxidation is 3×10^{-5} S m^{-1} (2×10^{-3} S m^{-1} before oxidation). Supplementary Figure 22b shows the Fe (*2p*) core level XPS spectrum for the oxidized MOF, which presents the coexistence of two Fe ions (Fe^{2+}/Fe^{3+}). The ratio of Fe^{2+}/Fe^{3+} is calculated to be ~1/3 (the ratio was ~2/1 before oxidation, shown in the manuscript). From this contrast experiment, we can infer that the conductivity dropped rapidly after oxidation, due to the increasing content of Fe^{3+}.

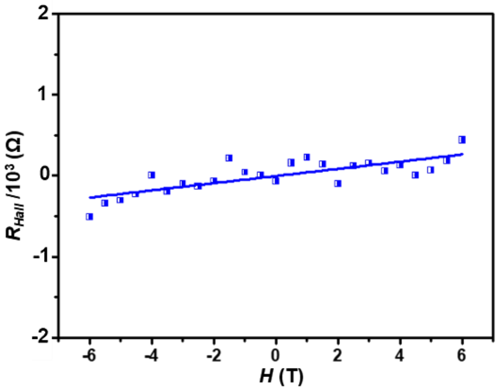

Supplementary Figure 22 | Magnetic field dependence of the Hall resistance (R_{Hall}) at 300 K, indicating the *p*-type semiconductor of K_3Fe_2[PcFe-O_8] (H: applied magnetic field). Dot: experimental results; Line: fitting results.

As shown in Supplementary Fig. 22, the fitting slope of the Hall effect shows a *p*-type semiconducting behavior in K_3Fe_2[PcFe-O_8], with the hole concentration of about 2.4×10^{14} cm^{-3} (calculated via $n_s = \frac{IB}{eV_H}$, where I is the current, **B** is the magnetic field, V_H is the Hall voltage and e is the elementary charge). The corresponding mobility is about 0.1 cm^2/Vs, where the carrier mobility is calculated through $\mu = \frac{1}{en_sR_s}$. However, the carrier mobility from the Hall measurement is much lower than its expected value, due to the contact resistance and the grain boundaries/gaps of K_3Fe_2[PcFe-O_8] particles in the pressed pellets. To overcome the above disadvantages, time-resolved THz spectroscopy (TRTS) was employed to estimate the nature of charge mobility without the contact resistance, which provided a mobility as high as 15±2 cm^2·V^{-1}·s^{-1}.

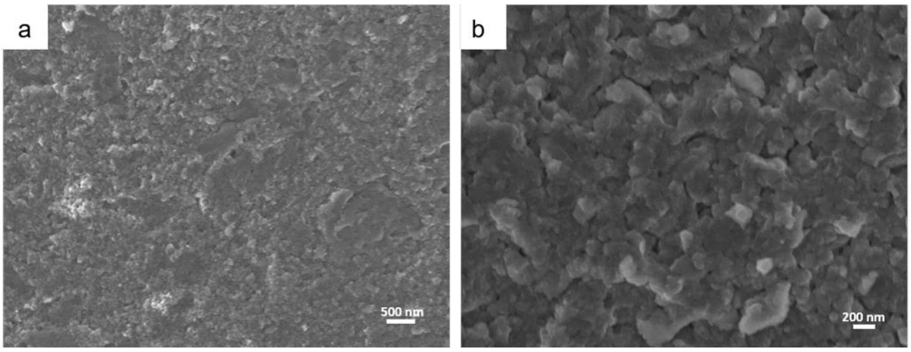

Supplementary Figure 23 | SEM images for pressed pellets of $K_3Fe_2[PcFe\text{-}O_8]$ with different magnification.

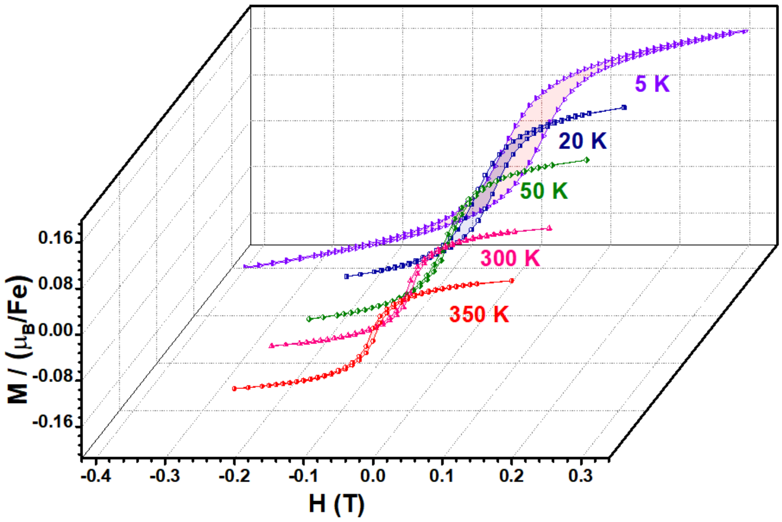

Supplementary Figure 24 | Enlarged M-H curves of $K_3Fe_2[PcFe\text{-}O_8]$ at different temperatures showing hysteresis loops.

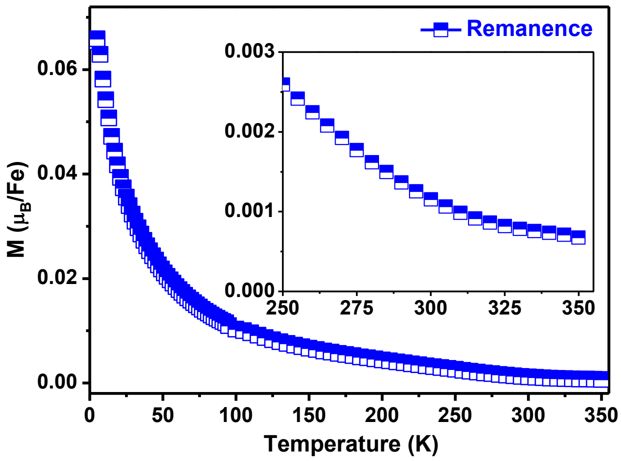

Supplementary Figure 25 | The remnant magnetization of K_3Fe_2[PcFe-O_8] obtained at *H* = 0 as a function of temperature. Insert: magnified curve at 250 K-350 K.

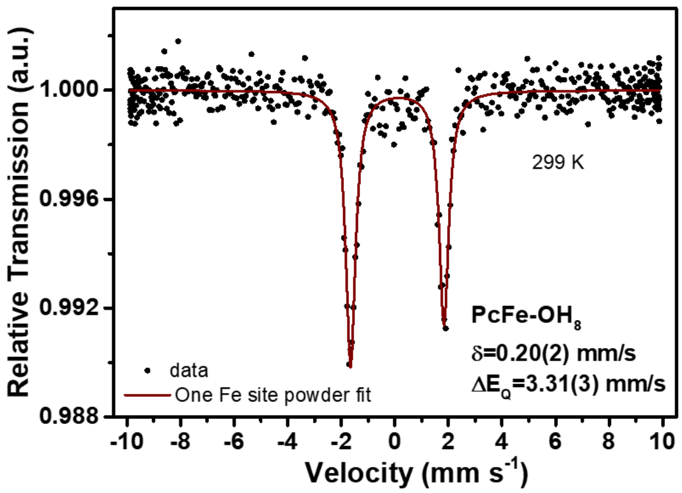

Supplementary Figure 26 | ^{57}Fe Mössbauer spectra of $PcFe\text{-}OH_8$ at 299 K.

Supplementary Tables

Supplementary Table 1 | Representative ferromagnetic semiconductors.

**Name**	**Shape**	**Growth Methods**	**Band gap**	***T_c***	***Ref.***
(Ga, Mn) As	Film	Low-temperature molecular beam epitaxy	1.4 eV	159K	[19]
CdMnTe	Film	Molecular beam epitaxy	——	< 3 K	[20]
$(Ga_{1-x},Mn_x)N$	Film	Molecular beam epitaxy	3.4 eV	13 K	[21]
$(Ba_{1-x}K_x)(Zn_{1-y}Mn_y)_2As_2$	Bulk	Arc-melting solid-state reaction	——	180 K	[22]
CrI_3	Monolayer sheet	Mechanical exfoliation of bulk crystals	——	45 K	[23]
CrI_3	2D Bulk material	Chemical vapor transport	1.2 eV	68 K	[24]
$Cr_2Ge_2Te_6$	Six-layers sheets	Mechanical exfoliation via adhesive tape	——	~10 K	[25]
$Cr_2Ge_2Te_6$	2D Bulk material	Heating in a sealed evacuated quartz tube	1.0 eV	61 K	[26]

Supplementary Table 2 | Different *Tc* values of currently reported MOFs at ambient pressure

Sample Name	Shape	*Magnetic ordering temperature*	*Ref.*
**$K_3Fe_2[PcFe\text{-}O_8]$**	**Polycrystals**	**>350 K**	**This**
$[Co_3^{II}(lac)2(pybz)_2]\cdot 7H_2O$	Crystals	32 K	27
$[Ni_7(C_4H_4O_4)_4(OH)_6(H_2O)_3]\cdot 7H_2O$	Crystals	20 K	28
SURMOF-2	Crystals	>20 K	29
$(Me_2NH_2)[Fe_2L_3]\cdot 2H_2O\cdot 6DMF$	Crystals	80 K	30
$(Me_2NH_2)[Fe_2L_3]$	Crystals	26 K	30
$(Cp_2Co)_{1.43}(Me_2NH_2)_{1.57}[Fe_2L_3]\cdot 4.9DMF$	Crystals	105 K	31
$(NBu_4)_2Fe_2(dhbq)_3$	Crystals	134 K	32
$Na_{0.9}(NBu_4)_{1.8}Fe_2(dhbq)_3$	Crystals	144 K	32
$[Ru_2(m\text{-fluorobenzoate})_4]_2(BTDA\text{-}TCNQ)$	Crystals	107 K	33
$Mn^{II}(TCNE)_{3/2}(I)_{1/2}$	Crystals	171 K*	34
$Mn^{II}(TCNE)I(OH_2)$	Crystals	171 K	35
PTC-Fe	Polycrystals	<20 K	36
$CrCl_2(pyrazine)_2$	Crystals	55 K	37

Pc = Phthalocyanine; pybz = 4-pyridyl benzoate; lac = D- and L-lactate;
L = 2,5-dichloro-3,6-dihydroxy-1,4-benzoquinone; Cp_2Co = cobaltocene;
dhbq= 2,5-dioxidobenzoquinone/1,2-dioxi-do-4,5-semiquinone;
BTDA-TCNQ = bis(1,2,5-thiadiazolo)tetracyanoquinodimethane;
PTC = 1,2,3,4,5,6,7,8,9,10,11,12-perthiolated coronene.
* ferrimagnetic transition temperature

Supplementary Table 3 | The electrochemical results of layered MOFs and the monomer

Sample Name	E_{ox1} (V vs SCE)	E_{red1} (V vs SCE)	HOMO eV	LUMO eV	$E_{g,cv}$ eV
**$K_3Fe_2[PcFe-O_8]$**	0.39	-0.24	-5.19	-4.56	0.63
**$PcFe-OH_8$**	0.35	-0.72	-5.15	-4.08	1.07

Supplementary Table 4 | Comparison of carrier mobility with those of other layer stacked coordination polymers and MOFs

Name	Sample Form	Mobility (cm^2/Vs)	Method	Reference
**$K_3Fe_2[PcFe\text{-}O_8]$**	Powder<br>Pellets	15±2<br>~0.1	TRTS<br>Van der Pauw	This work
**PiCBA**	Thin films	0.005	TRTS	38
**$Fe_3(THT)_2(NH_4)_3$**	Thin film	211±7<br>229±33	TRTS<br>Hall effect	39
**$Ni_3(HATP)_2$**	Thin Film	48.6	FET	40
**Cu-BHT**	Thin Film	116 (electrons)<br>99 (holes)	FET	41
**$K_xFe_2(BDP)_3$**	Single-crystalline particle	0.02(intrinsic)<br>0.29(reduction)	FET	42
**$Zn_2(TTFTB)$**	powder	0.2	FP-TRMC	43

THT = 2,3,6,7,10,11-hexathioltriphenylene; BHT = benzenehexathiol;

HATP = 2,3,6,7,10,11-hexaaminotriphenylene;

TRTS = time-resolved tetraherz spectroscopy; FET = field effect transistor;

FP-TRMC = flash photolysis-time-resolved microwave conductivity

Supplementary Table 5 | Fitting parameters of the Mössbauer spectra of $K_3Fe_2[PcFe\text{-}O_8]$.

The spectra in Fig. 4c-d and supplementary Fig. S26 are described by the following parameters: isomer shift *IS*, quadrupole splitting *QS*, line width Γ, the magnetic hyperfine field B_{hf} (here average of the B_{hf} distribution), the asymmetry parameter η ($0 \leq \eta \leq 1$) of the electric field gradient (efg), the polar angle Ω describing the relative orientation between the principal component V_{ZZ} of the efg and B_{hf}. For the magnetic hyperfine pattern *QS* is obtained as $QS = eQV_{ZZ}/2(1 + \eta^2/3)^{1/2}$, where Q is the

T (K)	*IS* ($mm \cdot s^{-1}$)	*QS* ($mm \cdot s^{-1}$)	η	B_{hf} (T)	ν_{fluc} (Mhz)	Γ ($mm \cdot s^{-1}$)	Area (%)
300	0.378(4)	0.843(6)	n.a.	0	n.a.	0.311(7)	58(1)
	0.242(10)	0	n.a.	30(5)	150(50)	0.29(6)	42(1)

quadrupole moment of the excited ^{57}Fe nucleus. Note that $\eta = 0$ is in agreement with a square planar coordination and $\Omega \sim 90°$ indicates that the spins are oriented in the FeN_4 plane.

References:

1. Ding, X. *et al.* Conducting metallophthalocyanine 2D covalent organic frameworks: the role of central metals in controlling π-electronic functions. *Chem. Commun.* **48**, 8952-8954 (2012).
2. Metz, J., Schneider, O. & Hanack, M. Synthesis and properties of substituted (phthalocyaninato)-iron and -cobalt compounds and their pyridine adducts. *Inorg. Chem.* **23**, 1065-1071 (1984).
3. Kresse, G. & Furthmüller, J. Efficiency of *ab*-initio total energy calculations for metals and semiconductors using a plane-wave basis set. *Comput. Mater. Sci.* **6**, 15-50 (1996).
4. Kresse, G. & Furthmüller, J. Efficient iterative schemes for *ab* initio total-energy calculations using a plane-wave basis set. *Phys. Rev. B* **54**, 11169-11186 (1996).
5. Blöchl, P. E. Projector augmented-wave method. *Phys. Rev. B* **50**, 17953-17979 (1994).
6. Kresse, G. & Joubert, D. From ultrasoft pseudopotentials to the projector augmented-wave method. *Phys. Rev. B* **59**, 1758-1775 (1999).
7. Perdew, J. P. *et al.* Atoms, molecules, solids, and surfaces: Applications of the generalized gradient approximation for exchange and correlation. *Phys. Rev. B* **46**, 6671-6687 (1992).
8. Perdew, J. P., Burke, K. & Ernzerhof, M. Generalized gradient approximation made simple. *Phys. Rev. Lett.* **77**, 3865-3868 (1996).
9. Vladimir, I. A., Aryasetiawan, F. & Lichtenstein, A. I. First-principles calculations of the electronic structure and spectra of strongly correlated systems: the LDA + U method. *J. Phys. Conden. Matter.* **9**, 767-808 (1997).
10. Li, W. *et al.* High temperature ferromagnetism in π-conjugated two-dimensional metal-organic frameworks. *Chem. Sci.* **8**, 2859-2867 (2017).
11. Monkhorst, H. J. & Pack, J. D. Special points for Brillouin-zone integrations. *Phys. Rev. B* **13**, 5188-5192 (1976).
12. Grimme, S. Semiempirical GGA-type density functional constructed with a long-range dispersion correction. *J. Comput. Chem.* **27**, 1787-1799 (2006).
13. Roeser, J. *et al.* Anionic silicate organic frameworks constructed from hexacoordinate silicon centres. *Nat. Chem.* **9**, 977-982 (2017).

14. D'Antonio, M. C. *et al.* Spectroscopic investigations of iron(II) and iron(III) oxalates. *J. Brazil. Chem. Soc.* **20**, 445-450 (2009).
15. Zheng, R. *et al.* A hollow spherical doped carbon catalyst derived from zeolitic imidazolate framework nanocrystals impregnated/covered with iron phthalocyanines. *J. Mater. Chem. A* **4**, 7859-7868 (2016).
16. Starr, D. E., Liu, Z., Hävecker, M., Knop-Gericke, A. & Bluhm, H. Investigation of solid/vapor interfaces using ambient pressure X-ray photoelectron spectroscopy. *Chem. Soc. Rev.* **42**, 5833-5857 (2013).
17. Ward, B., Chang, C. K. & Young, R. Spectral shifts upon reversible modifications of aldehyde (CHO) peripheral substituents in porphyrin, chlorin, and bacteriochlorin. A phenomenological explanation for the red shift of protonated Schiff base. *J. Am. Chem. Soc.* **106**, 3943-3950 (1984).
18. El-Nahass, M. M., Zeyada, H. M., Aziz, M. S. & Makhlouf, M. M. Optical absorption of tetraphenylporphyrin thin films in UV-vis-NIR region. *Spectrochimi. Acta. A* **61**, 3026-3031 (2005).
19. Edmonds, K. W. *et al.* Mn interstitial diffusion in (Ga,Mn)as. *Phys. Rev. Lett.* **92**, 037201 (2004).
20. Boukari, H. *et al.* Light and electric field control of ferromagnetism in magnetic quantum structures. *Phys. Rev. Lett.* **88**, 207204 (2002).
21. Stefanowicz, S. *et al.* Phase diagram and critical behavior of the random ferromagnet $Ga_{1-x}Mn_xN$. *Phys. Rev. B* **88**, 081201 (2013).
22. Zhao, K. *et al.* New diluted ferromagnetic semiconductor with Curie temperature up to 180 K and isostructural to the '122' iron-based superconductors. *Nat. Commun.* **4**, 1442, (2013).
23. Huang, B. *et al.* Layer-dependent ferromagnetism in a van der Waals crystal down to the monolayer limit. *Nature* **546**, 270-273 (2017).
24. McGuire, M. A., Dixit, H., Cooper, V. R. & Sales, B. C. Coupling of crystal structure and magnetism in the layered, ferromagnetic insulator CrI_3. *Chem. Mater.* **27**, 612-620 (2015).
25. Gong, C. *et al.* Discovery of intrinsic ferromagnetism in two-dimensional van der Waals crystals. *Nature* **546**, 265-269 (2017).

26. Carteaux, V., Brunet, D., Ouvrard, G. & Andre, G. Crystallographic, magnetic and electronic structures of a new layered ferromagnetic compound $Cr_2Ge_2Te_6$. *J. Phys. Condens. Matter.* **7**, 69-87 (1995).

27. Zeng, M.-H. *et al.* Nanoporous Cobalt(II) MOF exhibiting four magnetic ground states and changes in gas sorption upon post-synthetic modification. *J. Am. Chem. Soc.* **136**, 4680-4688 (2014).

28. Guillou, N., Livage, C., van Beek, W., Noguès, M. & Férey, G. A layered nickel succinate with unprecedented hexanickel units: atructure elucidation from powder-diffraction data, and magnetic and sorption properties. *Angew. Chem. Int. Ed.* **42**, 643-647 (2003).

29. Friedländer, S. *et al.* Linear chains of magnetic ions stacked with variable distance: ferromagnetic ordering with a curie temperature above 20 K. *Angew. Chem. Int. Ed.* **55**, 12683-12687 (2016).

30. Jeon Ie, R., Negru, B., Van Duyne, R. P. & Harris, T. D. A 2D semiquinone radical-containing microporous magnet with solvent-induced switching from T_c = 26 to 80 K. *J. Am. Chem. Soc.* **137**, 15699-15702 (2015).

31. DeGayner, J. A., Jeon, I.-R., Sun, L., Dincă, M. & Harris, T. D. 2D conductive iron-quinoid magnets ordering up to T_c = 105 K via heterogenous redox chemistry. *J. Am. Chem. Soc.* **139**, 4175-4184 (2017).

32. Darago, L. E., Aubrey, M. L., Yu, C. J., Gonzalez, M. I. & Long, J. R. Electronic conductivity, ferrimagnetic ordering, and reductive insertion mediated by organic mixed-valence in a ferric semiquinoid metal-organic framework. *J. Am. Chem. Soc.* **137**, 15703-15711 (2015).

33. Motokawa, N., Miyasaka, H., Yamashita, M. & Dunbar, K. R. An electron-transfer ferromagnet with T_c = 107 K based on a three-dimensional $[Ru_2]_2$/TCNQ system. *Angew. Chem. Int. Ed.* **47**, 7760-7763 (2008).

34. Stone, K. H. *et al.* $Mn^{II}(TCNE)_{3/2}(I_3)_{1/2}$-A 3D network-structured organic-based magnet and comparison to a 2D Analog. *Adv. Mater.* **22**, 2514-2519 (2010).

35. Lapidus, S. H., McConnell, A. C., Stephens, P. W. & Miller, J. S. Structure and magnetic ordering of a 2D $Mn^{II}(TCNE)I(OH_2)$ (TCNE = tetracyanoethylene) organic-based magnet (T_c = 171 K). *Chem. Commun.* **47**, 7602-7604 (2011).

36. Dong, R. *et al.* A coronene-based semiconducting twodimensional metal-organic framework with ferromagnetic behavior. *Nat. Commun.* **9**, 2637 (2018).

37. Pedersen, K. S. et al. Formation of the layered conductive magnet $CrCl_2(pyrazine)_2$ through redox-active coordination chemistry. *Nat. Chem.* **10**, 1056-1061 (2018).

38. Yang, C. et al. Coordination Polymer Framework Based On-Chip Micro-Supercapacitors with AC Line-Filtering Performance. *Angew. Chem. Int. Ed.* **56**, 3920-3924 (2017).

39. Dong, R. et al. High-mobility band-like charge transport in a semiconducting two-dimensional metal-organic framework. *Nat. Mater.* **17**, 1027-1032 (2018).

40. Wu, G., Huang, J., Zang, Y., He, J. & Xu, G. Porous Field-Effect Transistors Based on a Semiconductive Metal–Organic Framework. *J. Am. Chem. Soc.* **139**, 1360-1363 (2017).

41. Huang, X. et al. A two-dimensional *π-d* conjugated coordination polymer with extremely high electrical conductivity and ambipolar transport behaviour. *Nat. Commun.* **6**, 7408-7416 (2015).

42. Aubrey, M. L. et al. Electron Delocalization and Charge Mobility as a Function of Reduction in a Metal–Organic Framework. *Nat. Mater.* **17**, 625–632 (2018).

43. Narayant, T. C., Miyahai, T., Seki, S. & Dincă, M. High Charge Mobility in a Tetrathiafulvalene-Based Microporous Metal–Organic Framework. *J. Am. Chem. Soc.* **134**, 12932-12935 (2012).
